# Supplementary material for: Effects of stigma on help-seeking behavior in mental health: A community-based study in Ghana’s Sekyere South District in the Ashanti region
Source: Glob Ment Health (Camb). 2025 Dec 17;13:e8. doi: 10.1017/gmh.2025.10118 (PMC12835938; doi:10.1017/gmh.2025.10118)
Supplement: Afriyie et al. supplementary material [file S2054425125101180sup001.docx]

# **Effects of Stigma on Help-Seeking Behavior in Mental Health: A Community-Based Study in Ghana’s Sekyere South District in the Ashanti Region**

**Introduction:** Thank you for your participation in this academic study. The questionnaire includes questions on a range of topics, some of which you may find personal or sensitive. You are free to skip any question or withdraw from the study at any time without penalty. All your responses will be kept strictly confidential.

**Instructions:** Please indicate your response by ticking (✓), circling (○), or filling in (■) the appropriate box or space provided.

**SECTION A: SOCIODEMOGRAPHIC CHARACTERISTICS**

1. **Age (years)**
2. Below 20 [ ] 2) 20-35 [ ] 3) 36-50 [ ] 4) 51-65 [ ] 5) Above 65 [ ]
3. **Gender**
4. Male [ ] 2) Female [ ]
5. **Religion**
6. Christian [ ] 2) Muslim [ ] 3) Traditionalist [ ]
7. **Level of Education**
8. Primary [ ] 2) JHS [ ] 3) Secondary [ ] 4) Tertiary [ ] 5) None [ ]
9. **Marital status**
10. Single [ ] 2) Married [ ] 3) Divorced [ ] 4) Widowed [ ] 5) Separated [ ]
11. **Occupation**
12. Health worker 2) Student 3) Trader 4) Business man/woman 5) Other type

**SECTION B: FORMS OF STIGMA**

This section gathers information on different forms of mental health-related stigma experienced by:

- Individuals living with a mental health condition
- Their caregivers or family members
- Mental health professionals

***Read each statement carefully. Using the scale provided below, please indicate your response. We encourage you to be as candid and accurate as possible.***

| **FORMS OF STIGMA** | **1 = Not at all**  **2 = Rarely**  **3 = Sometimes**  **4 = Often**  **5 = Very Often** |
| --- | --- |
| 1. How often have you been stigmatized as a mental health worker, or a caregiver of a mentally ill patient or as a mentally ill patient? |  |
| 1. Insults from members in your community, school, workplace, or church |  |
| 1. Are mentally ill patients, or the caregivers or mental health workers refused   Employment opportunities? |  |
| 1. Loss of financial support |  |
| 1. Loss of self-esteem |  |
| 1. Verbal abuse |  |
| 1. Family blamed, ridiculed & mocked |  |
| 1. Perceived as "mean" person |  |
| 1. No access to clothing |  |
| 1. Loss of accommodation |  |
| 1. Loss of prospects of workplace promotion |  |
| 1. Refusal of employment |  |
| 1. Loss of job |  |
| 1. Exclusion from usual family activities |  |
| 1. Spouse desertion |  |
| 1. Loss of friends |  |
| 1. Employer discrimination |  |
| 1. Colleagues discrimination |  |
| 1. Withdrawal from school |  |
| 1. Denial of school admission |  |
| 1. Denial of or delay in medical treatment |  |

**SECTION C: DRIVERS OR FACILITATORS OF MENTAL HEALTH RELATED STIGMA**

This section collects data on the factors that drive or facilitate the stigmatization of people with mental health conditions.

**Instructions:** Using the 5-point agreement scale below, please indicate your level of agreement with each statement.

| **DRIVERS OR FACILITATORS OF MENTAL HEALTH**  **RELATED STIGMA** | **1 = Strongly Agree**  **2 = Agree**  **3 = Neutral**  **4 = Disagree**  **5= Strongly disagree** |
| --- | --- |
| 1. Fear of infection through casual contact for communicable diseases |  |
| 1. Presence or absence of occupational safety standards and protective supplies in health facilities |  |
| 1. Belief that those with mental illnesses are inferior |  |
| 1. Perception that people with mental health problems personally responsible for their illness |  |
| 1. Perception that people with mental health problems are incompetent and unable to work |  |
| 1. Belief that peoples with mental health problems are dangerous and unpredictable |  |
| 1. Lack of public knowledge or awareness about mental health conditions |  |
| 1. Cultural or religious beliefs that attribute mental illness to spiritual causes |  |

**SECTION D: ATTITUDES TOWARDS SEEKING PROFESSIONAL PSYCHOLOGICAL HELP SCALE** **(ATSPPHS)**

This scale is based on a four-point Likert scale with responses ranging from disagree to agree with five of the items reversed. Higher scores indicate more positive attitudes towards seeking professional help (total score ranges from 10 to 40).

*Instructions: Read each statement carefully and indicate your degree of agreement using the scale below. In responding, please be completely candid (1 = Disagree, 2 = Partly disagree, 3 = Partly agree, 4 = Agree)*

1. If I believed I was having a mental breakdown; my first inclination would be to get professional attention. [ ]
2. (R)The idea of talking about problems with a psychologist strikes me as a poor way to get rid of emotional conflicts. (R) [ ]
3. If I were experiencing a serious emotional crisis at this point in my life, I would be confident that I could find relief in psychotherapy. [ ]
4. There is something admirable in the attitude of a person who is willing to cope with his or her conflicts and fears without resorting to professional help. (R) [ ]
5. I would want to get psychological help if I were worried or upset for a long period of time. [ ]
6. I might want to have psychological counseling in the future. [ ]
7. A person with an emotional problem is not likely to solve it alone; he or she is likely to solve it with professional help. [ ]
8. Considering the time and expense involved in psychotherapy, it would have doubtful value for a person like me. (R) [ ]
9. A person should work out his or her own problems; getting psychological counseling would be a last resort. (R) [ ]
10. Personal and emotional troubles, like many things, tend to work out by themselves. (R) [ ]

*(R) = Reverse-scored item. Total ATSPPHS Score (sum of items 1–10, with reverse scoring applied): ________ / 40. (Total score ranges from 10 to 40).*
